# Genetic variation and cryptic lineage among the sergestid shrimp *Acetes americanus* (Decapoda)

**DOI:** 10.7717/peerj.14751

**Published:** 2023-02-13

**Authors:** Sabrina Morilhas Simões, Rogério Caetano Costa, Fabricio Lopes Carvalho, Abner Carvalho-Batista, Sarah de Souza Alves Teodoro, Fernando L. Mantelatto

**Affiliations:** 1 Department of Biological Sciences, Faculty of Sciences, University of State of São Paulo (UNESP), Bauru, São Paulo, Brazil; 2 Department of Biology, Faculty of Philosophy, Science and Letters at Ribeirao Preto (FFCLRP), University of São Paulo (USP), Ribeirão Preto, São Paulo, Brazil; 3 Federal University of Southern Bahia (UFSB), Ilhéus, Bahia, Brazil; 4 Paulista University (UNIP), Bauru, Bauru, São Paulo, Brazil; 5 Rio Grande Federal University (FURG), Rio Grande, Rio Grande do Sul, Brazil

**Keywords:** Crustacea, Molecular phylogeny, Western Atlantic, 16S rDNA, Sergestidae, Cytochrome Oxidase I (COI), Sergestoidea, Phylogenetics

## Abstract

The taxonomic status of the sergestid shrimp, *Acetes americanus,* has been questioned for several decades. No specific study has been performed thus far to resolve the incongruences. This species has a wide geographical range in the western Atlantic and is represented by two formally accepted subspecies: *Acetes americanus carolinae*, distributed in North America, and *Acetes americanus americanus*, present in South America. However, there are regions where the coexistence of both subspecies has been reported, such as Central America. This study aimed to genetically compare specimens of *A. a. americanus* collected in South America with *A. a. carolinae* sampled in North America to check for possible differences and the existence of more than one subspecies of *A. americanus* on the Brazilian coast. Based on the sequences of two informative markers, the cytochrome oxidase I region (COI) and 16S rRNA, phylogenetic reconstruction demonstrated well-defined clades with high support values, reinforcing the idea that *A. a. americanus* is genetically different from *A. a. carolinae*. Our hypothesis was corroborated as the specimens collected in Brazil were divided into two distinct lineages: the first composed of *A. a. americanus sensu stricto* (Brazil 1) and the second by *Acetes americanus* (Brazil 2). The three groups evidenced in the haplotype network were the same as those observed in the phylogenetic tree. The morphometric character (height/length of the thelycum) was effective in distinguishing *A. a.* Brazil 1 from *A. a. carolinae*. However, more detailed and conclusive studies comprising other characteristics to propose and describe a possible new entity are necessary. To the best of our knowledge, for the first time, the results of this study provide some insights into the taxonomic status of the sergestid shrimp *A. americanus* in the western Atlantic.

## Introduction

Genus *Acetes* H. Milne Edwards, 1830 comprises small planktonic shrimps (Wong, 2013), which are essential components of marine systems (Xiao & Greenwood, 1993). For a long time, the genus remained poorly understood among decapods concerning phylogeny. Recently, species have been contextualized in a global phylogeny (Vereshchaka, Lunina & Olesen, 2016a; Vereshchaka, 2017). Fourteen species of *Acetes* are recognized worldwide (De Grave & Fransen, 2011; WoRMS, 2022). Three species occur in the western Atlantic: *Acetes americanus* Ortmann, 1893; *Acetes marinus* (Omori, 1975); and *Acetes paraguayensis* Hansen, 1919.

Historically, four subspecies of *A. americanus* have been identified: *A. a. carolinae* Hansen, 1933 (type locality: Cove Beaufort, South Carolina, USA), *A. a. louisianensis* (Burkenroad, 1934) (type locality: Louisiana coast, from the west of the Mississippi River to Timbalier Island, Gulf of Mexico, USA), *A. a. limonensis* (Burkenroad, 1934) (type locality: Sweetwater River mouth, Panama), and *A. a. americanus* (Ortmann, 1893) (type locality: Tocantins River mouth, Brazil) (Burkenroad, 1934).

However, the subspecies *A. a. louisianensis* and *A. a. limonensis* presented intermediate characteristics of the other two subspecies. Therefore, they were traditionally considered clinal variants, which are not considered valid (Holthuis, 1948). Currently, *A. a. louisianensis* and *A. a. limonensis* are accepted as synonyms for *A. a. americanus* (WoRMS, 2022).

The existing taxonomy considers only *A. a. carolinae* and *A. a. americanus* as valid subspecies (Holthuis, 1948). Although these two subspecies are very similar, careful examination reveals minute morphological differences, as the body and cornea lengths of the southern representatives (*A. a. americanus*) are slightly larger than those of the northern representatives (*A. a. carolinae*) (Omori, 1975). Taxonomic inconsistencies in the subspecies of *A. americanus* have been reported since the 1970s (Omori, 1975). However, this remains unsolved.

**Figure 1:**
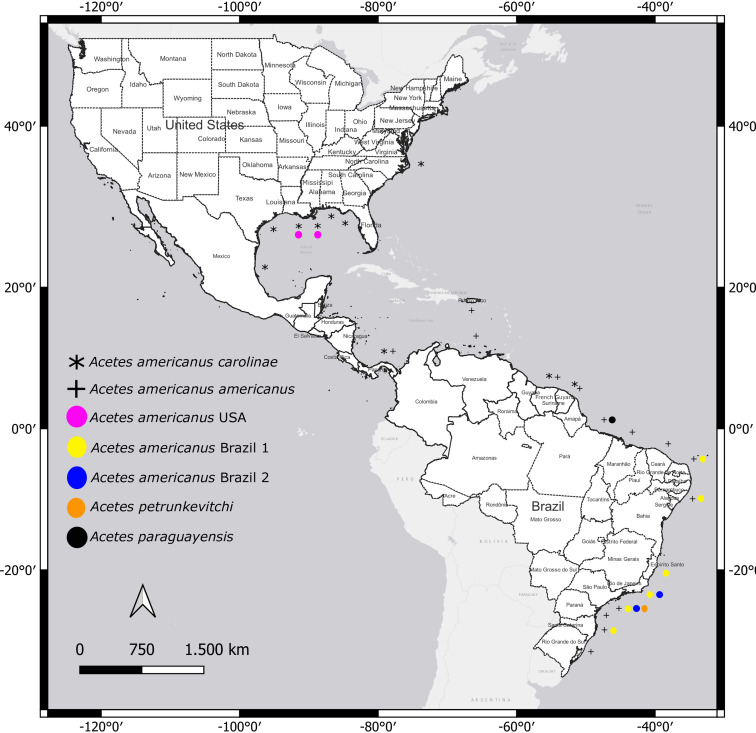
Distribution of studied species of *Acetes* in the Western Atlantic. Geographic distribution of the *Acetes americanus carolinae* (*) (Omori, 1975) and *Acetes americanus americanus* (+) (D’Incao and Martins, 2000). The highlighted circles indicate the sites studied by molecular analysis, *Acetes americanus* USA (pink), *Acetes americanus* Brazil 1 (yellow), *Acetes americanus* Brazil 2 (blue), *Acetes petrunkevitchi* (orange) and *Acetes paraguayensis* (black). Map created by authors using Qgis 3.24.3 (https://www.qgis.org/en/site/index.html).

*Acetes americanus carolinae* is distributed from North Carolina, Florida to the Gulf of Mexico, Panama, Suriname, and French Guiana (Omori, 1975); *A. a. americanus* Ortmann, 1893 occurs in Puerto Rico, Panama, Venezuela, Suriname, French Guiana, and Brazil (D’Incao & Martins, 2000; Mantelatto et al., 2022) (Fig. 1).

The uncertainty regarding the difference in the extant subspecies results from: (a) insufficient information about the subspecies’ habitats, especially where the two subspecies co-occur, (b) the small magnitude of morphological divergence among species of the genus *Acetes* and (c) the distribution.

Molecular tools were used to elucidate the taxonomic status of several marine shrimp species, whose morphology is insufficient to clarify their species identity (Carvalho, Magalhães & Mantelatto, 2014; Tavares & Gusmão, 2016; Carvalho, Magalhães & Mantelatto, 2020). Combined with morphological data, genetic data allowed the interpretation of variability patterns along the distribution of some taxa (Silva, Mesquita & Paula, 2010; Terossi & Mantelatto, 2012; Rossi & Mantelatto, 2013; Teodoro et al., 2016; Carvalho-Batista et al., 2019; França et al., 2019; Terossi & Mantelatto, 2020; França et al., 2021). The mitochondrial genes cytochrome c oxidase I (COI) and 16S rRNA have been considered powerful markers for molecular analysis in several studies of decapod shrimp at species and population levels (Gusmão, Lazoski & Solé-Cava, 2000; Maggioni et al., 2001; Lavery et al., 2004; Vergamini, Pileggi & Mantelatto, 2011; Terossi & Mantelatto, 2012; Teodoro et al., 2016; Carvalho-Batista et al., 2018; França et al., 2019).

In this study, we compared the *A. a. americanus* specimens collected from South America with those of *A. a. carolinae* sampled in North America using COI and 16S rRNA markers to test the genetic validity of both subspecies and the possible existence of other entities distributed along the western Atlantic.

## Materials & Methods

### Sampling of the biological material

The specimens used in this study were obtained through samplings and loans. All individuals of *A. americanus* from Penha/Santa Catarina, Ubatuba, Cananéia, São Vicente/São Paulo, Macaé/Rio de Janeiro, Baía Formosa/Rio Grande do Norte, and *A. petrunkevitchi* from Ubatuba/São Paulo were sampled using a fishing boat equipped with an otter trawl net with 2 m opening and 3 m in length. The mesh diameter (interknot distance) was five mm in the first half of the net and two mm in the final part. The obtained specimens were collected under field permit approval by Instituto Chico Mendes de Biodiversidade/ICMBio, number 23008-1 and Permanent License to FLM 11777-2) and deposited in the Crustacean Collection of the Department of Biology of the Faculty of Philosophy, Sciences, and Letters of Ribeirão Preto of the University of São Paulo, Brazil (CCDB/FFCLRP/USP) and in the Crustacean Collection of the Laboratory of Biology of Marine and Freshwater Shrimp, UNESP, Bauru, Brazil (CCLC/FC/UNESP) (Table 1 and Table S1).

**Table 1:** Size dimension relationship of females *Acetes americanus*. Sample size and ratio between height and length of the third thoracic sternite of the females of *Acetes americanus* Ortmann, 1893.

**Subspecies**	**Number of specimens**	**Locality (Latitud)**	**Catalog number**	**Average ± Standard Deviation**
*Acetes americanus* USA	28	Off Beaufort Inlet, North Carolina, United States (34 °N)	USNM74550	0.64 ± 0.08
*Acetes americanus* USA	2	Cape Lookout, North Carolina, United States (34°N)	USNM258701	0.65 ± 0.07
*Acetes americanus* USA	6	Beaufort, North Carolina, United States (34°N)	YPM005386	0.48 ± 0.08
*Acetes americanus* USA	5	Off mouth of North Edisto River, South Carolina, United States (32°N)	USNM258707	0.60 ± 0.11
*Acetes americanus* USA	9	Louisiana, Gulf of Mexico, United States (29°N)	YPM005387	0.57 ± 0.08
*Acetes americanus* USA	17	Laguna de Términos, Frente el Faro de Xicalango, México (19°N)	CNCR2402	0.56 ± 0.11
*Acetes americanus* Brazil 1	6	Channel at mouth of Bay, Puerto Rico (18°N)	USNM134695 to USNM134697	0.24 ± 0.04
*Acetes americanus* Brazil 1	17	Playa de Guayanes, Puerto Yabucoa, Puerto Rico (18°N)	USNM186645 to USNM186647	0.22 ± 0.06
*Acetes americanus* Brazil 1	20	Off Surinam Coast, Suriname (6°N)	USNM103101 to USNM103106	0.29 ± 0.08
*Acetes americanus* Brazil 1	26	Maceió,AL, Brazil (09°S)	MZUSP21210	0.38 ± 0.08
*Acetes americanus* Brazil 1	35	Macaé, RJ, Brazil (22°S)	CCLC0254	0.36 ± 0.07
*Acetes americanus* Brazil 1	28	Ubatuba, SP, Brazil (23°S)	CCLC0253	0.40 ± 0.34
*Acetes americanus* Brazil 1	13	Rio Grande, RS, Brazil (32°S)	MZUSP 9079	0.33 ± 0.07
*Acetes americanus* Brazil 2	35	Macaé, RJ, Brazil (22°S)	CCLC0261	0.79 ± 0.19
*Acetes americanus* Brazil 2	34	Cananéia, SP, Brazil (25°S)	CCLC0262	1.03 ± 0.29

**Notes.**

CCLC Crustacean Collection of the Laboratory of Biology of Marine and Freshwater Shrimp, UNESP, Bauru, Brazil MZUSP Zoology Museum of the University of São Paulo, São Paulo, Brazil USNM National Museum of Natural History, Smithsonian Institution, United States CNCR National Crustacean Collection, UNAM, Mexico YPM Peabody Museum of Natural History Yale University, United States

All other specimens used in this study were loaned from scientific collections: Zoology Museum of the University of São Paulo, São Paulo, Brazil (MZUSP); National Museum of Natural History, Smithsonian Institution, United States (USNM); National Museum of the Federal University of Rio de Janeiro (MNRJ); Oceanographic Museum of the University of Pernambuco, Brazil (MOUFPE); Federal University of Rio Grande (FURG); Crustacean Collection from the Department of Biology of the Faculty of Philosophy, Sciences and Letters of Ribeirão Preto, University of São Paulo (CCDB); Collection of Crustaceans of the Federal University of Sergipe (UFS); Collection of Crustaceans from the Federal University of Espírito Santo (UFES); Collection of Crustaceans from the PUCRS Museum of Science and Technology (MCP); Carcinological Collection of the Institute of Scientific and Technological Research of the State of Amapá, Macapá, AP (IEPA); National Crustacean Collection, UNAM, Mexico (CNCR); Peabody Museum of Natural History Yale University, United States (YPM); University of Louisiana at Lafayette, USA (ULLZ); and Zoological Museum Kiel, Germany (ZMK) (Table 1 and Table S1).

Specimens were morphologically identified following the identification key proposed by Omori (1975), D’Incao & Martins (2000), Vereshchaka, Lunina & Olesen (2016a), and Vereshchaka, Olesen & Lunina (2016b), using the shape of the third thoracic sternite (thelycum) in females and the petasma shape in males.

The names currently used for extant taxa follow the most recent literature (Vereshchaka, Lunina & Olesen, 2016a; Vereshchaka, Olesen & Lunina, 2016b) and are currently adopted by WoRMS (2022). Genus *Peisos* is dealt with differently. A review of the shrimp genera *Acetes*, *Peisos*, and *Sicyonella* (Vereshchaka, Lunina & Olesen, 2016a) proposed to include *Peisos* within the genus *Acetes* using morphological and phylogenetic information. Later, in a global phylogeny, Vereshchaka (2017) proposed new families, including Acetidae, to accommodate all species of *Acetes*. WoRMS (2022) does not adopt the proposed changes, that is, *Peisos* is an accepted genus, and Acetidae does not exist. We can conjecture a lack of taxonomic reassignment with a proper nomenclatural act in the previous paper. Considering that we were not able to find a clear justification and to avoid taxonomic instability, we maintained the proposed taxonomic status of *Peisos* as part of *Acetes* following Vereshchaka, Lunina & Olesen (2016a).

### DNA extraction and amplification

DNA extraction from the muscular abdominal tissue was performed following Mantelatto, Robles & Felder (2007); Mantelatto et al. (2009a); Mantelatto et al. (2009b) and Pileggi & Mantelatto (2010), with specific modifications (Carvalho-Batista et al., 2014). The regions of interest were amplified by the polymerase chain reactions (PCR) using the primers described in Table S2. Primers specific for this region were designed due to the difficulty in the amplification of COI (see Mantelatto et al., 2016 for details) (Table S2).

PCR reactions were performed with a total volume of 25 µL, containing 5 µL of betaine (5M) (Acros Organics), 4 µL of dNTPs (5 mM), 3 µl of MgCl_2_ (25 mM), 3 µl of 10X Taq buffer with KCl (Thermo Scientific), 2 µl of 1% bovine albumin (Sigma), 1 µl of each primer (10 µM), 1 µl of resuspended DNA (50 ng/ ml), and 0.5 µl of recombinant Taq DNA Polymerase (Thermo Scientific). The missing volume (25 µl) was filled with ultrapure water. PCR was performed using an Applied Biosystems^©^ Veriti 96 well thermal cycler. PCR steps comprised an initial denaturation period of 3 min at 95 °C, followed by 40 thermal cycles [30 s of denaturation at 95 °C, 45 s for annealing at variable temperature (42–44 °C for 16S; 44–50 °C for COI), 1 min for the extension at 72 °C, and final extension for 10 min at 72 °C. The obtained results were observed by 1.5% agarose gel electrophoresis and photographed with Olympus^©^ C-7070 digital camera in a UV M20 UV transilluminator.

The PCR products were purified using the Sureclean purification kit following the manufacturer’s protocol. The PCR-purified products were sequenced bidirectionally in automated sequencers (ABI 3100 Genetic Analyzer) at the Department of Technology of the Faculty of Sciences Agricultural and Veterinary Sciences of Jaboticabal, São Paulo State University.

The generated sequences were confirmed and edited (sequence consensus obtained from sense and antisense) in BioEdit 7.0.7.1 software (Hall, 1999) and aligned using CLUSTAL W (Thompson, Higging & Gibson, 1994).

Each genetic sample obtained for the analyses was deposited in the scientific collection of origin (Table S1).

### Molecular analyses

A total of 118 sequences used in this study were generated for this project. For the 16S rRNA region, 52 sequences of 518 bp were obtained, of which 29 were *A. a.* Brazil 1, nine were *A. a.* Brazil 2, five were *A. a. carolinae*, five were *A. paraguayensis*, and four were *A. petrunkevitchi*. For the COI region, 66 sequences of 589 bp were generated of which 36 were *A. a.* Brazil 1, 15 were *A. a.* Brazil 2, six were *A. a. carolinae*, six were *A. paraguayensis*, and three were *A. petrunkevitchi*.

However, an additional 20 sequences (COI) of *A. sibogae*, *A. japonicus*, *A. serrulatus*, *and A. indicus*, two sequences (16S and COI) of *A. petrunkevitchi*, and two sequences (16S and COI) of *B. faxoni* (outgroup) were retrieved from the GenBank database and used for phylogenetic and genetic distance analyses to complement this study (Table S1). As there are COI sequences for more *Acetes* species than 16S sequences available on GenBank, phylogenetic analysis with the COI gene generated a larger number of clades.

The mean nucleotide composition and genetic distances were estimated using MEGA 5.0 software (Tamura et al., 2011) and the Neighbor-Joining dendrogram based on the Kimura 2-parameter substitution model (Kimura, 1980).

The appropriate models of nucleotide evolution HKY + G for 16S and TPM1uf + G for COI were selected by Bayesian information criterion (BIC) in jModeltest 2.1.4 (Darriba et al., 2012). Selected models and estimated parameters (Table S3) were implemented in the Bayesian inferences and considered for the choice of the closest models in the maximum likelihood analyses. *Belzebub faxoni* (Borradaile, 1915) (Superfamily Sergestoidea, Family Luciferidae) was included as an outgroup following the most recent global phylogeny (Vereshchaka, Lunina & Olesen, 2016a; Vereshchaka, 2017). Bayesian inference was used to reconstruct the phylogenetic relationships of the species analyzed, with the two genes as distinct partitions in MrBayes *v.* 3.2.2 (Ronquist et al., 2012). Bayesian inference was performed with 30 million generations in two independent analyses, with five parallel chains each, one “cold” and four “hot”. The parameters were saved every 1,000 simulations. The analysis was completed on attaining stationarity (mean standard deviation <0.01) after the stipulated number of generations. The first quarter of the parameters and trees were discarded as burn-in (Ronquist, Van Der Mark & Huelsenbeck, 2009). The support values of the branches were obtained using the a *posteriori* probability method.

This analysis was performed for the 16S and COI genes separately and a concatenated matrix of both the trees was generated and edited in the program Figtree v.1.3.1 (Rambaut, 2007).

### Population analyses

Only the COI gene was used for population analyses. The use of this gene has been shown to be efficient in decapod population studies (Gusmão, Lazoski & Solé-Cava, 2005; Laurenzano, Mantelatto & Schubart, 2013; Rossi & Mantelatto, 2013; Carvalho-Batista et al., 2014; Teodoro et al., 2015) because it is considered to be more variable (Schubart & Huber, 2006).

The number of haplotypes was calculated in the DnaSP 4.10.9 software (Rozas & Rozas, 1999). Haplotype networks were constructed using the median-joining method, using the network program (Bandelt, Forster & Röhl, 1999), based on the data prepared in the DnaSP. Haplotype and nucleotide diversities, analysis of molecular variance (AMOVA), and pairwise fixation indices (Fst) (Excoffier, Smouse & Quattro, 1992) were calculated using Arlequin 3.11 (Excoffier, Laval & Schneider, 2005).

### Morphometric analyses

We used only females (281 individuals) of *A. americanus* for morphometric analyses. Previous observations focused on the difference in size and shape of the female thelycum between the subspecies, motivating us to obtain potential information to complement our study.

Individuals were sexed based on the presence of petasma (first pleopod) in males and the thelycum (third thoracic sternite) in females (Xiao & Greenwood, 1993).

Morphometric measurements were obtained using a stereo microscope Zeiss^©^ Stemi 2000C connected to an imaging system Zeiss^©^ AxioVision, with an error of up to 0.01 mm. We measured the height (HT) and length (LT) of the thelycum and carapace length (CL).

Following the methodology proposed by Marramà & Kriwet (2017), data were standardized by calculating the ratio between each measure and carapace length (CL), removing the effect of size. The log transformation of data (Log (X+1)) was used to overcome the issue of the non-normal distribution of data by unstretching large-scale values. Moreover, log transformation is useful to considerably reduce variation due to ontogeny (allometric effect) since we assumed that specimens of different developmental stages were subjected to our analyses. A Euclidean distance matrix was constructed using the log-transformed data. Multivariate analysis of variance (PERMANOVA) was applied to test similarities within subspecies and localities (*P* < 0.005) (Anderson, 2001) using PRIMER software (version 6; Clarke & Gorley, 2006). A Permanova pairwise post-hoc test was performed to further investigate the differences between the subspecies (*P* < 0.005). Principal component analysis (PCA) was performed to characterize the differences between groups using the measured parameters (HT/CL and LT/CL ratios). Similarity percentage tests (SIMPER) were used to evaluate which measure contributed more to the differentiation between subspecies.

## Results

### Morphological identification of taxonomic entities

**Figure 2:**
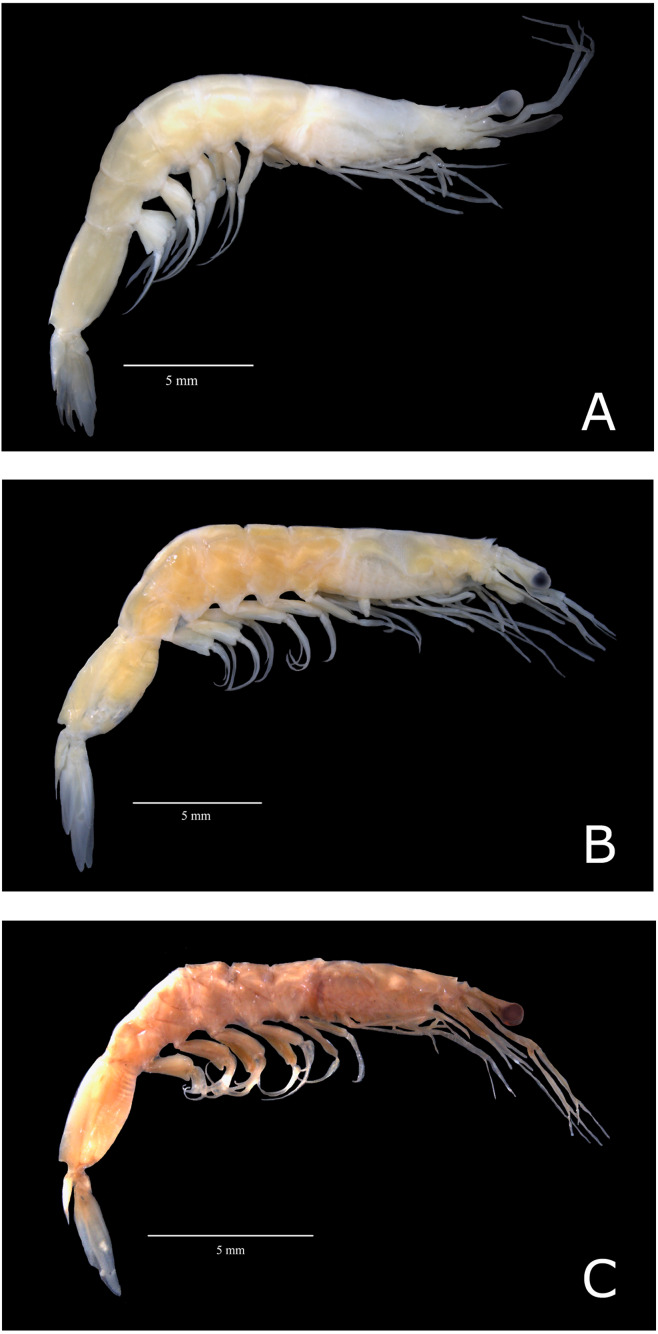
Color photos of estudied species of *Acetes*. Lateral view of *Acetes americanus* Ortmann, 1893. (A) Female of the *Acetes americanus* Brazil 1 (CCLC 0260); (B) female of the *Acetes americanus* Brazil 2 (CCLC 0255); (C) male of the *Acetes americanus* USA (CCLC 0268). CCLC: Crustacean Collection of the Laboratory of Biology of Marine and Freshwater Shrimp. Photo credit: Régis Augusto Pescinelli.

All specimens loaned from the United States of America presented characteristics of the subspecies *A. a. carolinae* (hereafter named *A. a.* USA) (Fig. 2C). However, Brazilian samples fell into two categories: (i) samples exhibiting characteristics of the subspecies *A. a. americanus* senso stricto (hereafter named “*A. a.* Brazil 1”; Fig. 2A) and (ii) samples that exhibited morphological divergence from the nominal subspecies (hereafter named “*A. a.* Brazil 2” (Fig. 2B)). The sampling localities of *A. a.* Brazil 1 and *A. a.* Brazil 2 are shown in Fig. 1.

### Genetic distance

16S rRNA gene: The intraspecific distances varied from 0% (*A. a.* USA, *A. a.* Brazil 2, and *A. petrunkevitchi*) to 0.20% (*A. paraguayensis*) (Table 2). The interspecific distances between congeneric species varied from 0.99 to 11.8%. The distance to the outgroup was 25.9 to 28.5% (Table 3). Regarding the *A. americanus* subspecies, the lowest interspecific distance was between *A. a.* Brazil 2 and *A. a.* USA (0.99%). The highest was *A. a.* Brazil 1 and *A. a.* USA (2.26%) (Table 3).

COI gene: The intraspecific distances varied from 0.02% (*A. a.* Brazil 1) to 0.97% (*A. paraguayensis*) (Table 2). The interspecific distances between the congeneric species varied from 4.86 to 22.3%. The distance to the outgroup was 21.8 to 25.5% (Table 3). Regarding the *A. americanus* subspecies, the lowest interspecific distance was between *A. a.* Brazil 1 and *A. a.* USA (4.86%). The highest was *A. a.* Brazil 2 and *A. a.* USA (8.08%) (Table 3).

### Phylogenetic analyses

**Figure 3:**
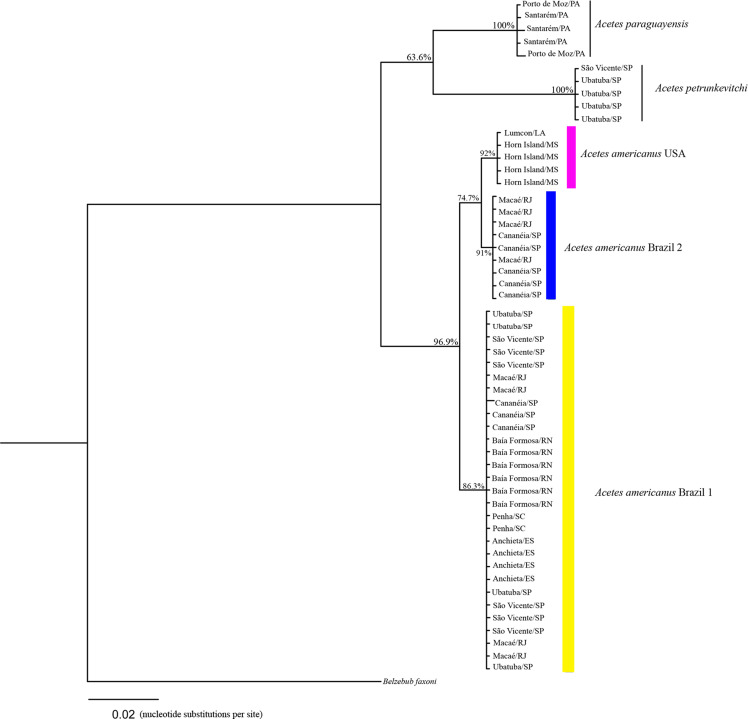
Phylogenetic reconstruction of *Acetes* based on 16mt marker. Phylogenetic tree of Bayesian inference for the *Acetes* species based on the 16S region with Bayesian posterior probabilities indicated (only posterior probabilities > 50% are shown).

The 16S rRNA phylogenetic analyses showed the following clades (Fig. 3): Clade 1, formed by *A. paraguayensis* sampled in north Brazil (Santarém and Porto de Moz/Pará); Clade 2, formed by *A. petrunkevitchi* sampled in southeast Brazil (São Vicente and Ubatuba/São Paulo); Clade 3, formed by *A. a.* USA sampled in the United States (Lumcon/LA and Horn Island/MS); Clade 4, formed by *A. a.* Brazil 2 sampled in southeast Brazil (Cananéia/São Paulo and Macaé/Rio de Janeiro); Clade 5, formed by *A. a.* Brazil 1 sampled in the south (Penha/Santa Catarina), southeast (Ubatuba, São Vicente and Cananéia/São Paulo, Macaé/ Rio de Janeiro, Anchieta/Espirito Santo) and northeast Brazil (Baía Formosa/Rio Grande do Norte).

**Table 2:** Intraspecific genetic distance from 16S and COI genes. Average intraspecific genetic distance (%) from 16S and Cytochrome Oxidase 1 (COI) gene ± standard deviation.

**Subspecies and species**	**16S**	**COI**
	**Genetic distance** **(± standard deviation)**	**Genetic distance** **(± standard deviation)**
*Acetes americanus* USA	0.00 (± 0.000)	0.18 (± 0.001)
*Acetes americanus* Brazil 1	0.01 (± 0.001)	0.02 (± 0.000)
*Acetes americanus* Brazil 2	0.00 (± 0.000)	0.26 (± 0.001)
*Acetes paraguayensis*	0.20 (± 0.0012)	0.97 (± 0.003)
*Acetes petrunkevitchi*	0.00 (± 0.000)	0.26 (± 0.001)

**Table 3:** Interspecific distance from 16S and COI genes between species of *Acetes*. Matrix of average interspecific distance (%) from 16S (below) and Cytochrome Oxidase 1 (COI) gene (above) between species of *Acetes* (numbers on top) ± standard deviation (values on bottom).

	*Acetes americanus* Brazil 1	*Acetes americanus* Brazil 2	*Acetes americanus* USA	*Acetes paraguayensis*	*Acetes petrunkevitchi*	*Belzebub faxoni*
*Acetes americanus* Brazil 1		6.44 (± 0.011)	4.86 (± 0.010)	22.1 (± 0.023)	19.0 (± 0.019)	21.8 (± 0.021)
*Acetes americanus* Brazil 2	1.97 (± 0.006)		8.08 (± 0.013)	22.0 (± 0.023)	20.2 (± 0.020)	22.9 (± 0.021)
*Acetes americanus* USA	2.26 (± 0.007)	0.99 (± 0.004)		23.3 (± 0.023)	21.6 (± 0.022)	23.7 (± 0.022)
*Acetes paraguayensis*	7.93 (± 0.012)	8.38 (± 0.013)	8.04 (± 0.013)		21.4 (± 0.021)	25.5 (± 0.021)
*Acetes petrunkevitchi*	11.8 (± 0.016)	11.1 (± 0.015)	11.4 (± 0.015)	10.0 (± 0.015)		25.4 (± 0.019)
*Belzebub faxoni*	26.6 (± 0.026)	25.9 (± 0.026)	26.3 (± 0.027)	28.5 (± 0.028)	27.5 (± 0.026)	

**Figure 4:**
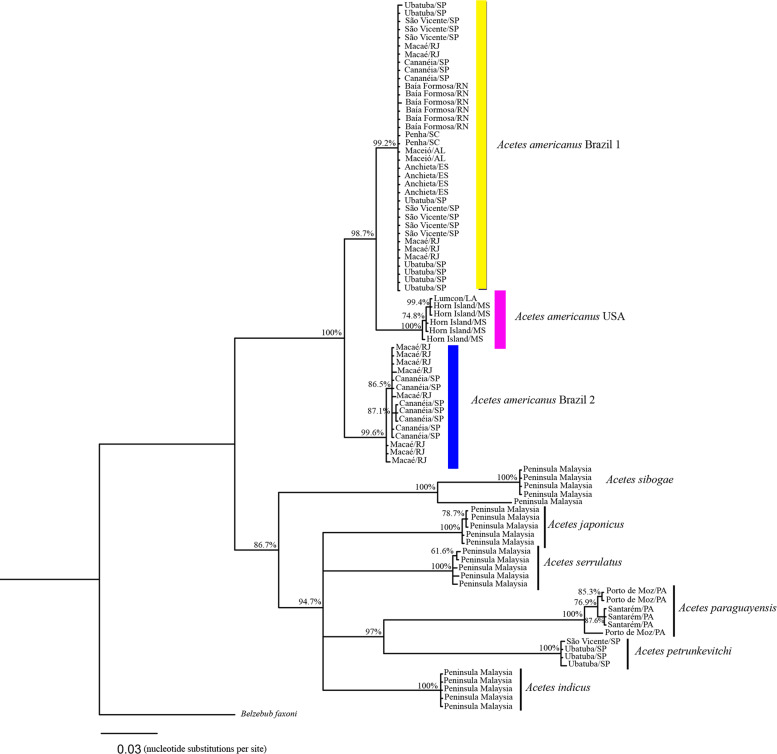
Phylogenetic reconstruction of *Acetes* based on COI marker. Phylogenetic tree of Bayesian inference for the *Acetes* species based on the COI region with Bayesian posterior probabilities indicated (only posterior probabilities > 50% are shown).

Based on the COI sequences, the phylogenetic analysis revealed the following clades (Fig. 4): Clade 1, formed by *A. a.* Brazil 1 sampled in the south (Penha/Santa Catarina), southeast (Ubatuba and Cananéia/São Paulo, Macaé/Rio de Janeiro, Anchieta/Espirito Santo) and northeast Brazil (Baía Formosa/Rio Grande do Norte, Maceió/Alagoas); Clade 2, formed by *A. a.* USA sampled in the United States (Lumcon/LA and Horn Island/MS); Clade 3, formed by *A. a.* Brazil 2 sampled in southeast Brazil (Cananéia/SP and Macaé/RJ); Clade 4, formed by *A. sibogae* sampled in Peninsula Malaysia; Clade 5, formed by *A. japonicus* sampled in Peninsula Malaysia; Clade 6, formed by *A. serrulatus* sampled in Peninsula Malaysia; Clade 7, formed by *A. paraguayensis* sampled in north Brazil (Santarém and Porto de Moz/Pará); Clade 8, formed by *A. petrunkevitchi* sampled in southeast Brazil (Ubatuba/SP); Clade 9, formed by *A. indicus* sampled in Peninsula Malaysia.

**Figure 5:**
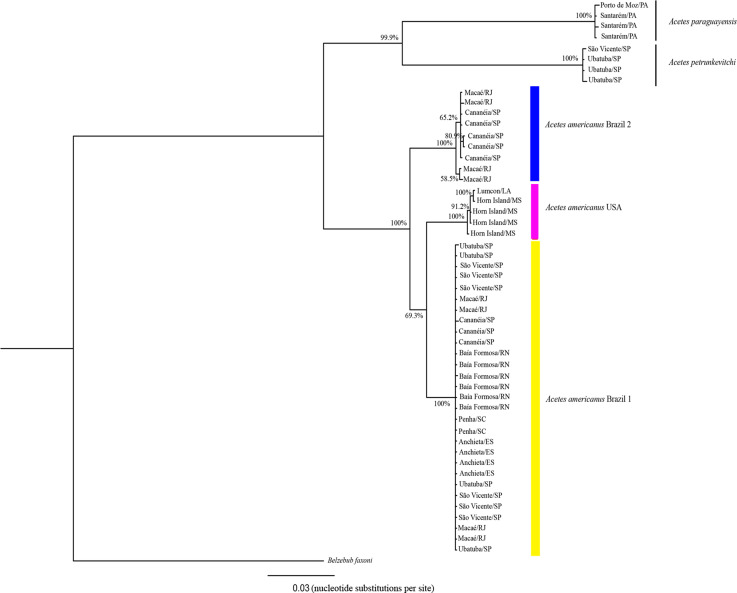
Pylogenetic reconstruction of *Acetes* based on concatenated markers. Phylogenetic tree of Bayesian inference for the *Acetes* species based on 16S and COI concatenated data with Bayesian posterior probabilities indicated (only posterior probabilities > 50% are shown).

The phylogenetic tree constructed based on the concatenated data (16S rRNA and COI) generated the same clades observed in the phylogenetic tree constructed with 16S rRNA and COI separately. In addition, *A. a.* USA and *A. a.* Brazil 2 were sister clades (Fig. 5), whereas COI, *A. a.* USA was a sister clade of *A. a.* Brazil 1 (Fig. 4).

The phylogenetic trees constructed (16S rRNA, COI, and concatenated data) resulted in the formation of two distinct clades of *A. americanus* sampled in Brazil with a high support value, in contrast to the clade formed by *A. a. carolinae* sampled in the United States. Furthermore, the *A. americanus* group appears to be a sister taxon to all other *Acetes* (Fig. 4).

### Population analyses

**Figure 6:**
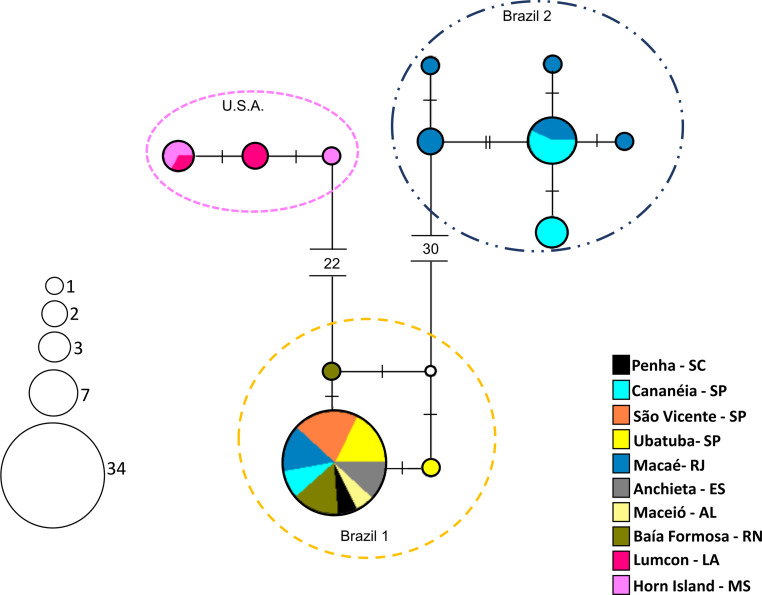
Haplotype network (COI region) showing the groups of *Acetes*. Haplotype network (COI region) according to a median-joining analysis, indicating the distribution of 12 haplotypes of *Acetes americanus* Ortmann, 1893. The size of the circle of each haplotype is proportionate to its frequency in the sample. Each small dash represents a mutational step.

The haplotype network exhibited a genetic structure in the three groups, corresponding to those observed in the phylogenetic trees (Fig. 6). Available COI sequences of *A. americanus* (*N* = 57) resulted in 12 haplotypes of which six were unique, that is, represented by a single individual. “Brazil 1″presented the lowest haplotype (*h* = 0.11) and nucleotide (*π* = 0.00022) diversities. This group included 34 individuals sharing one haplotype and two individuals with unique haplotypes. “USA” and “Brazil 2” presented similar high haplotype (*h* = 0.73 and *h* = 0.76, respectively) and nucleotide diversities (*π* = 0.00181 and *π* = 0.00277, respectively).

Analysis of molecular variance (AMOVA) did no detect genetic structuring of any subspecies among the location studied (*p* > 0.05) (Table 4).

### Morphometric analysis

The ratio between the height and length of the thelycum was determined for several western Atlantic locations (Table 1). The ratio values were higher in *A. a.* USA and *A. a.* Brazil 2 than in *A. a.* Brazil 1 (Table 1). The studied *A. americanus* groups (BR1, BR2, and the USA) were morphologically different (*P* = 0.0002) (Table 5). Permanova Pairwise tests indicated that at least one of the taxonomic entities is different from the others (*P* = 0.0001) (Table 6). However, when comparing *A. americanus* groups with each other, Pairwise tests indicated that there was a statistically significant difference only between Brazil 1 and Brazil 2 (*t* = 3.1563; *p* = 0.0018).

**Figure 7:**
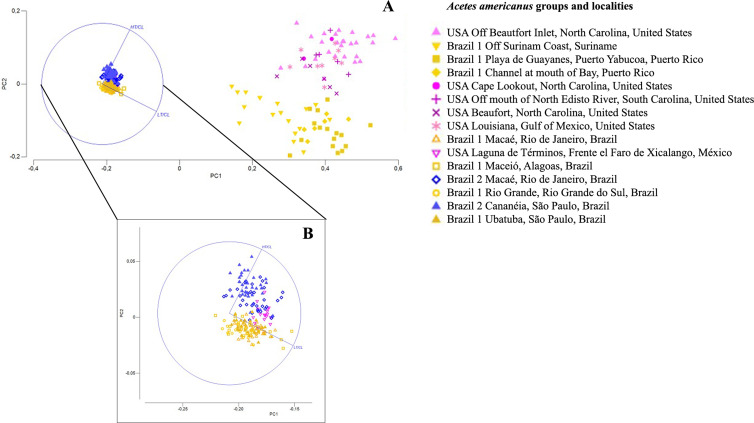
Principal component analysis depicting the morphometric groups of *Acetes*. (A) Principal Component Analysis (PCA) of the variation in thelycum morphology of the *Acetes americanus* groups (*A. americanus* USA, *A. americanus* Brazil 1 and *A. americanus* Brazil 2); (B) An enlarged area of the image “A”.

The PCA visualization plot shows that *A. a.* Brazil 1 is more similar to *A. a.* Brazil 2 than to *A. a.* USA (Fig. 7). A simplified Simper test showed that the LT/CL RATIO was responsible for the differences between subspecies, contributing more than 73% for Brazil 1, more than 53% for Brazil 2 and more than 61% for the USA (Table 7; Fig. 7).

## Discussion

Our analyses revealed the existence of three lineages of *A. americanus*: *A. a.* Brazil 1 *sensu stricto*, *A. a.* Brazil 2, and *A. a.* USA*.* Through molecular analysis, we were also able to identify and contextualize another species of *Acetes* that occurs in Brazil, *A. paraguayensis*. Therefore, mitochondrial DNA can be considered an efficient tool for solving taxonomic identification of the genus *Acetes* at species level.

One of the species concepts widely accepted in systematics (Tsoi, Wang & Chu, 2005) is defined as a group of mating individuals or having the potential for it differing from other groups because they are reproductively isolated (Mayr, 1942). However, there are several alternative concepts of species (De Queiroz, 2007). The haplotype network results suggest that there are three possible lineages for *A. americanus*: *A. a.* Brazil 1 *sensu stricto*, *A. a.* Brazil 2, and *A. a.* USA. Population-based analyses of mitochondrial DNA indicate that entities are reproductively isolated when gene flow is low (Tsoi, Wang & Chu, 2005). The low level of gene flow is essential evidence for speciation (Futuyma, 1998). Therefore, the non-sharing of haplotypes found in this study indicates that *A. a.* Brazil 1, *A. a.* Brazil 2, and *A. a.* USA are genetically distinct, with a low gene flow between them.

**Table 4:** Molecular variance with specimens of *Acetes americanus*. Analysis of molecular variance (AMOVA) performed with specimens of *Acetes americanus*.

		*Acetes americanus* Brazil 1	*Acetes americanus* Brazil 2	*Acetes americanus* USA
Variation source (%)	Among locations	−14.82	8.65	−12.50
	Within locations	114.82	91.35	112.50
FST (P)		−0.1482 (0.967)	0.0865 (0.152)	−0.125 (1.000)

**Notes.**

* Significant values, *P* < 0.05.

**Table 5:** Permanova analysis of *Acetes americanus* groups. Results of Permanova analysis considering *Acetes americanus* groups (BR1, BR2 and USA).

**Source**	**Df**	**SS**	**MS**	**Pseudo-F**	**P(perm)**	**perms**
Subspecies	1	1.3898E−2	1.3898E−2	7.1277	0.0058	9927
Locality	12	13.679	1.1399	584.6	0.0001	9909
Res	266	0.51867	1.9499E−3			

**Notes.**

D.f. degrees of freedom SS sum of squares MS mean square Pseudo-F statistic P(perm) probability Perms permutations performed

**Table 6:** Permanova Pairwise test of *Acetes americanus* groups. Results of the Permanova Pairwise test considering *Acetes americanus* groups (BR1, BR2 and USA).

**Source**	**df**	**SS**	**MS**	**Pseudo-F**	**P(perm)**	**Unique perms**
Subspecies	2	7.843	3.9215	76.786	0.0001	9949
Res	278	14.198	5,11E+02			
Total	280	22.041				

**Notes.**

D.f. degrees of freedom SS sum of squares MS mean square Pseudo-F statistic P(perm) probability Perms permutations performed

**Table 7:** Results of similarity percentages analysis of body dimensions. Results of SIMPER (Similarity Percentages) analysis.

Species	Average Abundance	Average Similarity	Sim/SD	Contrib%	Cum.%
**Group** ***Acetes americanus carolinae*** **(USA)**					
**Average similarity: 67.12**					
LT/CL ratio	0.46	41.37	2.03	61.64	61.64
HT/CL ratio	0.30	25.75	1.89	38.36	100.00
**Group** ***Acetes americanus*** **”BRAZIL 1”**					
**Average similarity:** **63.52**					
LT/CL ratio	0.24	46.81	1.91	73.69	73.69
HT/CL ratio	0.08	16.71	1.89	26.31	100.00
**Group** ***Acetes americanus*** **”BRAZIL 2”**					
**Average similarity:** **89.83**					
LT/CL ratio	0.07	47.63	7.97	53.03	53.03
HT/CL ratio	0.06	42.20	7.71	46.97	100.00

**Notes.**

CL Carapace Length HT height of the third thoracic sternite LT length of the third thoracic sternite SD Standard Deviation

The *A. a.* Brazil 1 individuals sampled in several Brazilian regions (northeast: RN, AL, SE; southeast: ES, RJ, SP; south: SC) shared haplotypes between them, indicating the existence of gene flow among these populations. Recent studies on other decapod crustaceans have also shown genetic homogeneity among populations sampled along the western Atlantic, along the Brazilian coast (Laurenzano, Farias & Schubart, 2012; Terossi & Mantelatto, 2012; Rossi & Mantelatto, 2013; Wieman et al., 2013; Laurenzano, Mantelatto & Schubart, 2013; Carvalho-Batista et al., 2014; Teodoro et al., 2015; Nishikawa, Negri & Mantelatto, 2021). A potential reason for population homogeneity can be ascribed to the high dispersion capacity of planktonic larvae and the absence of barriers to gene flow. Although nothing is known about the larval dispersal of *A. americanus*, the dispersal power is high for species of the genus *Acetes*, as they exhibit long planktonic larval stages (∼6 weeks) before becoming juveniles and adults (Rao, 1968). This premise has also been proposed for the other decapods tested (see references above).

The levels of genetic divergence (COI) among congeneric species of the crustaceans may vary up to 17%, a high value compared to other animal groups (Costa et al., 2007). Lepidopteran insects have a genetic divergence among congeneric species of only 6.1% (Hebert, Ratnasingham & Waard, 2003). Bird species show a variation of 7.93% (Hebert et al., 2004a), and fish have a 9.93% divergence (Ward et al., 2005). For sergestid shrimp, the rate of genetic divergence (COI) was also high. The genetic divergence found between *A. indicus*, *A. serrulatus*, *A. japonicus*, and *A. sibogae* ranged from 14.6 to 20.47% (Wong, 2013).

Our results indicated genetic divergence values (COI) from 4.86 to 8.08% between the *A. americanus* subspecies, which are low when compared with the studies mentioned above. However, if we compare these results to those of studies of cryptic or closely related shrimp species, these values are similar. Species morphologically similar displayed genetic divergences of 2.4 to 7% and were considered different (Gusmão, Lazoski & Solé-Cava, 2000; Lavery et al., 2004; Tsoi, Wang & Chu, 2005; Lagrue et al., 2014). Carvalho-Batista et al. (2019) found higher genetic divergence values (up to 13.5%) among the genus Seabob *Xiphopenaeus*. However, among some closely related *Xiphopenaeus* species, the variation ranged from 2.7–3.3%.

The intraspecific distance (COI) (0.02–0.97%) was lower than the interspecific (4.86–22.3%) for *Acetes americanus* subspecies. This difference, known as the gap, is used by the DNA barcode technique to differentiate species (Hebert et al., 2004b; Ward, 2009; Carvalho-Batista et al., 2019), which reinforces that the three lineages analyzed can potentially be considered as different taxonomic entities, pending future morphological characterization. This study showed variation between *A. americanus* subspecies (0.99–2.26%) similar to the ones previously reported for penaeid shrimps (Costa et al., 2007; Silva, Mesquita & Paula, 2010).

Comparing only the measures of the height/length ratio of the female thelycum (Table 1), our results corroborated Omori (1975), which also found that this measurement differed between the two subspecies (*A. a. americanus* e *A. a. carolinae*). The values found by Omori (1975) for *A. a. carolinae* were 0.56–0.80 (mean 0.68) for North Carolina, 0.50–0.83 (0.66) for Louisiana and Texas, 0.70 and 0.53 for Panama and Suriname specimens, respectively, and values of 0.21–0.31 for specimens of *A. a. americanus* collected in Santos/SP. In our study, *A. a.* USA also presented higher values of height/length of the thelycum when compared to *A. a.* Brazil 1. However, we revealed the presence of *A. a.* Brazil 2 on the Brazilian coast showing high values of the height/length of the thelycum (Table 1). Therefore, if only this character is analyzed, the individual could be misidentified with *A. a.* USA.

PCA analysis showed a separation of the three subspecies groups (Brazil 1, Brazil 2 and USA) when considering thelycum measurements and the carapace length. In the transition areas where the subspecies occur, however, individuals from Suriname and Puerto Rico identified as *A.a.* Brazil 1 were close to *A. a.* USA, and individuals from Mexico identified as *A. a.* USA were close *A. a.* Brazil 1 and *A. a.* Brazil 2. These results point to an interesting geographic pattern, with the separation between individuals collected in the south (Terminos Mexico) and the north of the Gulf of Mexico - GOM (Louisiana). The two portions of the GOM have different water temperatures and have been separated into distinct biogeographic provinces by different authors (Boschi, 2000; Briggs & Bowen, 2012). Additionally, within the GOM, different cyclonic and anti-ciclonic flows occur separating the circulation in each locality (Schmitz Jr et al., 2005) which may be responsible for maintaining the isolation within each one of them. Therefore, this result can be associated with responses to the environmental conditions of the region, as phenotypic variations can be caused by both genetic information and environmental variations (Templeton, 2006). Considering these results, the molecular identification of the subspecies from these transition areas, using the protocols of this study, would be recommended to clarify this issue.

*Acetes americanus* Brazil 1 is genetically different from *A. a.* USA. In addition, the specimens sampled in Brazil formed two distinct clades: the first was composed of *A. a.* Brazil 1 and the second of *A. a.* Brazil 2. As stated earlier, *A. a.* Brazil 1 exhibits the diagnostic characteristics of *A. americanus americanus*, whereas *A. a.* Brazil 2 exhibits characteristics similar to those of *A. amecicanus carolinae.* Phylogenetic and population analyses pointed to the divergence of *A. a.* Brazil 2 from *A. a.* USA. Usually, a single characteristic to be fixed after reproductive isolation is sufficient for the diagnosis of a species (Mink & Sites Jr, 1996). However, no morphological characteristics that can discriminate these subspecies have been detected thus far. Several studies have shown that many species with few or no morphological characteristics are distinguished by genetic differences (Reuschel, Cuesta & Schubart, 2010; Puillandre et al., 2011; Carvalho, Magalhães & Mantelatto, 2014; Delić et al., 2017; Mandai et al., 2018). A study focusing on the morphological characteristics used to discriminate *A. americanus* in Brazil should clarify and provide more information about this new possible taxonomic entity. Furthermore, the hypothesis that *A. a.* Brazil 2 is a different entity, previously described (*A. a. louisianensis* or *A. a. limonensis*), cannot be disregarded.

### General phylogeny

A previous phylogenetic study of *Acetes* proposed that the *Acetes* clade without *A. petrunkevitchi* (former *Peisos petrunkevitchi*) never gained robust support, thus considering *Peisos* as a junior synonym of *Acetes* (Vereshchaka, Lunina & Olesen, 2016a). Although we carried out an analysis with a robust but limited number of samples, our results confirmed the phylogenetic positioning recovered by both 16S rRNA and COI genes and indicated that *A. petrunkevitchi* is part of the *Acetes* group, but in all analyses forming a single clade of “*Acetes paraguayensis* + *A. petrunkevitchi*”, with a high support value for 16S rRNA and concatenated data. As only mitochondrial genes were used, further studies adding nuclear markers should be carried out to test the topology recovered herein.

However, recent studies about the morphology of the male reproductive system and spermatophore of *A. petrunkevitchi* differed from those of *A. americanus*, *A. marinus* and *A. paraguayensis*, which remains open the discussion about the inclusion of *Peisos* in the *Acetes* group (Salti, 2020).

The global morphological phylogeny proposed for the superfamily Sergestoidea showed significant changes in taxonomy, with the description of three new families, particularly because the family Sergestidae was not considered monophyletic (Vereshchaka, 2017). As a result, *A. americanus* was classified in the new family Acetidae, as proposed by Vereshchaka (2017). The author proposed that a single clade *of A. marinus* and *A. paraguayensis* within Acetidae received high bootstrap support. Our concatenated molecular phylogeny indicated a close relationship between *A. paraguayensis* and *A. petrunkevitchi* in a separate clade, as mentioned above. Additional samples of these species, including *A. marinus*, confirm this hypothesis. This molecular reconstruction sheds light on the unsolved evolutionary relations between the species of the genus *Acetes*, which should be investigated using more comprehensive integrated studies and the addition of nuclear markers.

##  Supplemental Information

10.7717/peerj.14751/supp-1 Supplemental Information 1 Data on specimens of *Acetes* and outgroup species used in phylogenetic analyses Specimens of *Acetes* and outgroup species used in phylogenetic analyses, sampling locality, catalog number, primers and GenBank accession number.

10.7717/peerj.14751/supp-2 Supplemental Information 2 Primers used in molecular analysis Information on the primers used in the present study.

10.7717/peerj.14751/supp-3 Supplemental Information 3 Models of nucleotide evolution for 16S rRNA and COI Models of nucleotide evolution selected in jModeltest for 16S rRNA and cytochrome c oxidase subunit I (COI) genes based on the Bayesian information criterion.

10.7717/peerj.14751/supp-4 Supplemental Information 4 Original sequences 16S

10.7717/peerj.14751/supp-5 Supplemental Information 5 COI Original sequences

## Additional Information and Declarations

### Field Study Permissions

The following information was supplied relating to field study approvals (i.e., approving body and any reference numbers): All specimens used in our analysis were collected under Federal autorization from the Instituto Chico Mendes de Biodiversidade/ICMBio (#11777-2, #23008-1).

### DNA Deposition

The following information was supplied regarding the deposition of DNA sequences: The group of mitochondrial (16S and COI) sequences are available at GenBank: OP035650–OP035701 (16S) and OP060465–OP060530 (COI).

### Data Availability

The following information was supplied regarding data availability: The raw data is available in the Supplemental Files.
