# Supplementary material for: Genetic variation and cryptic lineage among the sergestid shrimp Acetes americanus (Decapoda)
Source: PeerJ. 2023 Feb 13;11:e14751. doi: 10.7717/peerj.14751 (PMC9933772; doi:10.7717/peerj.14751)
Supplement: Supplemental Information 1 — Specimens of Acetes and outgroup species used in phylogenetic analyses, sampling locality, catalog number, primers and GenBank accession number. [file peerj-11-14751-s001.docx]

| Specimens | Locality | Catalogue Number | GenBank accession numbers | | References |
| --- | --- | --- | --- | --- | --- |
|  |  |  | 16S | COI | Present work |
| *Acetes americanus* Brazil 1 | Ubatuba/SP/Brazil | CCLC 0253 | OP035650 | OP060465 | Present work |
| *Acetes americanus* Brazil 1 | Ubatuba/SP/Brazil | CCLC 0253 | OP035651 | OP060466 | Present work |
| *Acetes americanus* Brazil 1 | São Vicente/SP/Brazil | CCDB 4939 | OP035652 | OP060467 | Present work |
| *Acetes americanus* Brazil 1 | São Vicente/SP/Brazil | CCDB 4939 | OP035653 | OP060468 | Present work |
| *Acetes americanus* Brazil 1 | São Vicente/SP/Brazil | CCDB 4939 | OP035654 | OP060469 | Present work |
| *Acetes americanus* Brazil 1 | Macaé/RJ/Brazil | CCLC 0254 | OP035655 | OP060470 | Present work |
| *Acetes americanus* Brazil 1 | Macaé/RJ/Brazil | CCLC 0254 | OP035656 | OP060471 | Present work |
| *Acetes americanus* Brazil 1 | Cananéia/SP/Brazil | CCLC 0256 | OP035657 | OP060473 | Present work |
| *Acetes americanus* Brazil 1 | Cananéia/SP/Brazil | CCLC 0256 | OP035658 | OP060474 | Present work |
| *Acetes americanus* Brazil 1 | Cananéia/SP/Brazil | CCLC 0256 | OP035659 | OP060475 | Present work |
| *Acetes americanus* Brazil 1 | Baía Formosa/RN/Brazil | CCDB 6320 | OP035660 | OP060476 | Present work |
| *Acetes americanus* Brazil 1 | Baía Formosa/RN/Brazil | CCDB 6320 | OP035661 | OP060477 | Present work |
| *Acetes americanus* Brazil 1 | Baía Formosa/RN/Brazil | CCDB 6320 | OP035662 | OP060478 | Present work |
| *Acetes americanus* Brazil 1 | Baía Formosa/RN/Brazil | CCDB 6320 | OP035663 | OP060479 | Present work |
| *Acetes americanus* Brazil 1 | Baía Formosa/RN/Brazil | CCDB 6320 | OP035664 | OP060480 | Present work |
| *Acetes americanus* Brazil 1 | Baía Formosa/RN/Brazil | CCDB 6320 | OP035665 | OP060481 | Present work |
| *Acetes americanus* Brazil 1 | Penha/SC/Brazil | CCLC 0257 | OP035666 | OP060482 | Present work |
| *Acetes americanus* Brazil 1 | Penha/SC/Brazil | CCLC 0257 | OP035667 | OP060483 | Present work |
| *Acetes americanus* Brazil 1 | Maceió/AL/Brazil | MZUSP 33010 | - | OP060484 | Present work |
| *Acetes americanus* Brazil 1 | Maceió/AL/Brazil | MZUSP 33012 | - | OP060485 | Present work |
| *Acetes americanus* Brazil 1 | Anchieta/ES/Brazil | CCLC 0258 | OP035668 | OP060486 | Present work |
| *Acetes americanus* Brazil 1 | Anchieta/ES/Brazil | CCLC 0258 | OP035669 | OP060487 | Present work |
| *Acetes americanus* Brazil 1 | Anchieta/ES/Brazil | CCLC 0259 | OP035670 | OP060488 | Present work |
| *Acetes americanus* Brazil 1 | Anchieta/ES/Brazil | CCLC 0259 | OP035671 | OP060489 | Present work |
| *Acetes americanus* Brazil 1 | Ubatuba/SP/Brazil | CCLC 0260 | OP035676 | OP060494 | Present work |
| *Acetes americanus* Brazil 1 | São Vicente/SP/Brazil | CCDB 4953 | OP035677 | OP060495 | Present work |
| *Acetes americanus* Brazil 1 | São Vicente/SP/Brazil | CCDB 4953 | OP035678 | OP060496 | Present work |
| *Acetes americanus* Brazil 1 | São Vicente/SP/Brazil | CCDB 4953 | OP035689 | OP060497 | Present work |
| *Acetes americanus* Brazil 1 | São Vicente/SP/Brazil | CCDB 4953 | - | OP060510 | Present work |
| *Acetes americanus* Brazil 1 | Macaé/RJ/Brazil | CCLC 0254 | OP035690 | OP060511 | Present work |
| *Acetes americanus* Brazil 1 | Macaé/RJ/Brazil | CCLC 0254 | OP035691 | OP060512 | Present work |
| *Acetes americanus* Brazil 1 | Macaé/RJ/Brazil | CCLC 0254 | - | OP060513 | Present work |
| *Acetes americanus* Brazil 1 | Ubatuba/SP/Brazil | CCLC 0264 | OP035694 | OP060515 | Present work |
| *Acetes americanus* Brazil 1 | Ubatuba/SP/Brazil | CCLC 0264 | - | OP060516 | Present work |
| *Acetes americanus* Brazil 1 | Ubatuba/SP/Brazil | CCLC 0266 | - | OP060519 | Present work |
| *Acetes americanus* Brazil 1 | Ubatuba/SP/Brazil | CCLC 0266 | - | OP060520 | Present work |
| *Acetes americanus* Brazil 2 | Macaé/RJ/Brazil | CCLC 0255 | - | OP060472 | Present work |
| *Acetes americanus* Brazil 2 | Macaé/RJ/Brazil | CCLC 0261 | OP035684 | OP060504 | Present work |
| *Acetes americanus* Brazil 2 | Macaé/RJ/Brazil | CCLC 0261 | OP035685 | OP060505 | Present work |
| *Acetes americanus* Brazil 2 | Macaé/RJ/Brazil | CCLC 0261 | OP035686 | OP060506 | Present work |
| *Acetes americanus* Brazil 2 | Macaé/RJ/Brazil | CCLC 0261 | - | OP060507 | Present work |
| *Acetes americanus* Brazil 2 | Cananéia/SP/Brazil | CCLC 0262 | OP035687 | OP060508 | Present work |
| *Acetes americanus* Brazil 2 | Cananéia/SP/Brazil | CCDB 3251 | OP035688 | OP060509 | Present work |
| *Acetes americanus* Brazil 2 | Macaé/RJ/Brazil | CCLC 0267 | OP035697 | OP060521 | Present work |
| *Acetes americanus* Brazil 2 | Macaé/RJ/Brazil | CCLC 0267 | - | OP060522 | Present work |
| *Acetes americanus* Brazil 2 | Macaé/RJ/Brazil | CCLC 0267 | - | OP060523 | Present work |
| *Acetes americanus* Brazil 2 | Cananéia/SP/Brazil | CCDB 3251 | OP035698 | OP060524 | Present work |
| *Acetes americanus* Brazil 2 | Cananéia/SP/Brazil | CCDB 3251 | OP035699 | OP060525 | Present work |
| *Acetes americanus* Brazil 2 | Cananéia/SP/Brazil | CCDB 3251 | OP035700 | OP060526 | Present work |
| *Acetes americanus* Brazil 2 | Cananéia/SP/Brazil | CCDB 3251 | - | OP060527 | Present work |
| *Acetes americanus* Brazil 2 | Cananéia/SP/Brazil | CCDB 3251 | - | OP060528 | Present work |
| *Acetes americanus* USA | Lumcon/Louisiana/EUA | ULLZ 15593 | OP035679 | OP060498 | Present work |
| *Acetes americanus* USA | Horn Island/Mississipi/EUA | ULLZ 14545 | OP035680 | OP060499 | Present work |
| *Acetes americanus* USA | Horn Island/Mississipi/EUA | ULLZ 14545 | OP035681 | OP060500 | Present work |
| *Acetes americanus* USA | Horn Island/Mississipi/EUA | ULLZ 14545 | OP035682 | OP060501 | Present work |
| *Acetes americanus* USA | Horn Island/Mississipi/EUA | ULLZ 14545 | OP035683 | OP060502 | Present work |
| *Acetes americanus* USA | Horn Island/Mississipi/EUA | ULLZ 14545 | - | OP060503 | Present work |
| *Acetes paraguayensis* | Xingu/PA/Brazil | CCDB 4791 | OP035672 | OP060490 | Present work |
| *Acetes paraguayensis* | Santarém/PA/Brazil | CCDB 6200 | OP035673 | OP060491 | Present work |
| *Acetes paraguayensis* | Santarém/PA/Brazil | CCDB 6200 | OP035674 | OP060492 | Present work |
| *Acetes paraguayensis* | Santarém/PA/Brazil | CCDB 6200 | OP035675 | OP060493 | Present work |
| *Acetes paraguayensis* | Xingu/PA/Brazil | CCDB 4791 | - | OP060529 | Present work |
| *Acetes paraguayensis* | Xingu/PA/Brazil | CCDB 4792 | OP035701 | OP060530 | Present work |
| *Acetes paraguayensis* | Xingu/PA/Brazil | CCDB 4792 | - | - | Present work |
| *Acetes petrunkevitchi* | Ubatuba/SP/Brazil | CCLC 0263 | OP035692 | - | Present work |
| *Acetes petrunkevitchi* | Ubatuba/SP/Brazil | CCLC 0263 | OP035693 | OP060514 | Present work |
| *Acetes petrunkevitchi* | Ubatuba/SP/Brazil | CCLC 0263 | OP035695 | OP060517 | Present work |
| *Acetes petrunkevitchi* | Ubatuba/SP/Brazil | CCLC 0265 | OP035696 | OP060518 | Present work |
| *Acetes sibogae* | Malaysia | - | - | HQ630587 | Wong, B.Y., Khoo, G. & Ong, A.H.K. (Unpublished) |
| *Acetes sibogae* | Malaysia | - | - | HQ630586 | Wong, B.Y., Khoo, G. & Ong, A.H.K. (Unpublished) |
| *Acetes sibogae* | Malaysia | - | - | HQ630585 | Wong, B.Y., Khoo, G. & Ong, A.H.K. (Unpublished) |
| *Acetes sibogae* | Malaysia | - | - | HQ630584 | Wong, B.Y., Khoo, G. & Ong, A.H.K. (Unpublished) |
| *Acetes sibogae* | Malaysia | - | - | HQ630579 | Wong, B.Y., Khoo, G. & Ong, A.H.K. (Unpublished) |
| *Acetes japonicus* | Malaysia | - | - | HQ630575 | Wong, B.Y., Khoo, G. & Ong, A.H.K. (Unpublished) |
| *Acetes japonicus* | Malaysia | - | - | HQ630574 | Wong, B.Y., Khoo, G. & Ong, A.H.K. (Unpublished) |
| *Acetes japonicus* | Malaysia | - | - | HQ630573 | Wong, B.Y., Khoo, G. & Ong, A.H.K. (Unpublished) |
| *Acetes japonicus* | Malaysia | - | - | HQ630572 | Wong, B.Y., Khoo, G. & Ong, A.H.K. (Unpublished) |
| *Acetes japonicus* | China | - | - | KF977240 | Zhang, *et al.* (Unpublished) |
| *Acetes serrulatus* | Malaysia | - | - | HQ630562 | Wong, B.Y., Khoo, G. & Ong, A.H.K. (Unpublished) |
| *Acetes serrulatus* | Malaysia | - | - | HQ630561 | Wong, B.Y., Khoo, G. & Ong, A.H.K. (Unpublished) |
| *Acetes serrulatus* | Malaysia | - | - | HQ630560 | Wong, B.Y., Khoo, G. & Ong, A.H.K. (Unpublished) |
| *Acetes serrulatus* | Malaysia | - | - | HQ630559 | Wong, B.Y., Khoo, G. & Ong, A.H.K. (Unpublished) |
| *Acetes serrulatus* | Malaysia | - | - | HQ630558 | Wong, B.Y., Khoo, G. & Ong, A.H.K. (Unpublished) |
| *Acetes indicus* | Malaysia | - | - | HQ630497 | Wong, B.Y., Khoo, G. & Ong, A.H.K. (Unpublished) |
| *Acetes indicus* | Malaysia | - | - | HQ630496 | Wong, B.Y., Khoo, G. & Ong, A.H.K. (Unpublished) |
| *Acetes indicus* | Malaysia | - | - | HQ630495 | Wong, B.Y., Khoo, G. & Ong, A.H.K. (Unpublished) |
| *Acetes indicus* | Malaysia | - | - | HQ630494 | Wong, B.Y., Khoo, G. & Ong, A.H.K. (Unpublished) |
| *Acetes indicus* | Malaysia | - | - | HQ630493 | Wong, B.Y., Khoo, G. & Ong, A.H.K. (Unpublished) |
| *Acetes petrunkevitchi* | Brazil | CCDB 4940 | KX196540 | KX196598 | Rossi, N. & Mantelatto, F.L. (Unpublished) |
| *Belzebub faxoni*  (outgroup) | Ubatuba/SP/Brazil | - | KY449064 | KY449077 | Carvalho-Batista *et al.*, 2019 |

CCLC: Crustacean Collection of the Laboratory of Biology of Marine and Freshwater Shrimp, UNESP, Bauru, Brazil; CCDB: Crustacean Collection of the Department of Biology of the Faculty of Philosophy, Sciences and Letters of Ribeirão Preto of the University of São Paulo, Brazil; MZUSP: Zoology Museum of the University of São Paulo, São Paulo, Brazil; ULLZ: University of Louisiana at Lafayette, USA
