# Supplementary material for: Genetic variation and cryptic lineage among the sergestid shrimp Acetes americanus (Decapoda)
Source: PeerJ. 2023 Feb 13;11:e14751. doi: 10.7717/peerj.14751 (PMC9933772; doi:10.7717/peerj.14751)
Supplement: Supplemental Information 2 — Information on the primers used in the present study. [file peerj-11-14751-s002.docx]

| **Primer** | **Gene** | **Sequence (5’- 3’)** | **References** |
| --- | --- | --- | --- |
| 1472-H2 | 16S | AGATAGAAACCAACCTGG | Crandall & Fitzpatrick 1996 |
| 16S-L2 | 16S | TGCCTGTTTATCAAAAACAT | Schubart, Cuesta & Felder 2002 |
| H9 | 16S | CCGGTCTGAACTCAGATCACGT | Schubart & Huber 2006 |
| L9 | 16S | CGCCTGTTTATCAAAAACAT | Schubart & Huber 2006 |
| COIAL1m | COI | GAGCTTGAGCYGGRATAGTAGG | Mantelatto *et al*. 2016 |
| COIAH1m | COI | CTCCWGCRGGGTCAAAGAAAGA | Mantelatto *et al*. 2016 |
| COIAL2o | COI | ACGCAACGATGATTATTTTCTAC | Mantelatto *et al.* 2016 |
| COIAH2o | COI | GACCAAAAAATCAGAATAAATGTTG | Mantelatto *et al.* 2016 |
| COIAH2m | COI | GACCRAAAAATCARAATAAATGTTG | Mantelatto *et al.* 2016 |
