# Supplementary material for: Genetic variation and cryptic lineage among the sergestid shrimp Acetes americanus (Decapoda)
Source: PeerJ. 2023 Feb 13;11:e14751. doi: 10.7717/peerj.14751 (PMC9933772; doi:10.7717/peerj.14751)
Supplement: Supplemental Information 3 — Models of nucleotide evolution selected in jModeltest for 16S rRNA and cytochrome c oxidase subunit I (COI) genes based on the Bayesian information criterion. [file peerj-11-14751-s003.docx]

|  | **16S rRNA** | **COI** |
| --- | --- | --- |
| Model | HKY + G | TPM1uf + G |
| Gama distribution | 0.2780 | 0.1970 |
| AC rate of substitution | 2.1748 | 1.0000 |
| AG rate of substitution | 1.0000 | 6.3376 |
| AT rate of substitution | 1.4813 | 7.6823 |
| CG rate of substitution | 1.0000 | 7.6823 |
| CT rate of substitution | 2.1748 | 27.9018 |
| GT rate of substitution | 1.0000 | 1.0000 |
| A frequency | 0.3482 | 0.3765 |
| C frequency | 0.1793 | 0.1207 |
| G frequency | 0.1208 | 0.1950 |
| T frequency | 0.3517 | 0.3078 |
